# Probing the Neural Basis of Perceptual Phenomenology with the Touch-Induced Visual Illusion

**DOI:** 10.1371/journal.pone.0047788

**Published:** 2012-10-23

**Authors:** Philip Servos, Allison Boyd

**Affiliations:** Department of Psychology, Wilfrid Laurier University, Waterloo, Ontario, Canada; University of Montreal, Canada

## Abstract

Using the touch-induced visual illusion we examine whether the brain regions involved in coding sensory information are dissociable from those that contain decision information. Activity in the intraparietal sulcus, as measured by functional magnetic resonance imaging, was associated with the illusion suggesting a sensory coding role whereas activity in the middle occipital gyrus differentially modulated activity according to the decisions made by subjects consistent with their reported perceptual phenomenology.

## Introduction

Over the past decade much progress has been made in elucidating the cortical regions involved in multisensory illusions such as the McGurk effect [1] and the rubber hand illusion [2]. One outstanding question, however, is whether or not such brain regions that appear to combine information from various sensory streams are also the regions involved in the perceptual phenomenology underlying these illusions. In other words, are the cortical regions of the brain involved in perceptual decision making dissociable from those that simply combine the information from the various sensory streams. We use a novel multisensory illusion, the touch-induced visual illusion [3], to answer this question.

In this illusion subjects are presented with either one or two flashes of a small disk in quick succession while simultaneously receiving two taps on their index finger and asked to report on the number of flashes they perceived. Even though subjects were instructed to ignore the number of taps they felt, one flash was erroneously perceived as two flashes on a substantial proportion of such trials. Critically, during trials in which subjects experienced the illusion their reported perceptual phenomenology was such that they were unable to distinguish these trials, in which only one visual flash was presented, from trials in which two visual flashes were presented.

What makes this paradigm ideal for our purpose is that we can examine the neural activity on trials in which subjects succumb to the illusion (i.e., respond ‘two’ when only one flash is presented) to trials in which they do not (i.e., respond ‘one’ when only one flash is presented or ‘two’ when two flashes are presented). We would expect different patterns of cortical activation on trials in which subjects experience the illusion relative to when they do not. Such differing patterns have been observed in unisensory illusions [4], [5]. Further, we would expect the brain regions involved in processing the multisensory stimulus attributes to be dissociable from the brain regions involved in perceptual decision making based on monkey neurophysiological evidence in the visual and tactile systems [6], [7] as well as human modeling work of visual perception [8]. We predict that a region involved in the decision making process should not differentiate between illusion trials (one-flash trials perceived as two flashes) and veridical two-flash trials whereas a multisensory region involved in binding the visual and tactile streams prior to decision making might be expected to differentially process illusion and non-illusion trials.

## Methods

### Ethics Statement

This research was approved by the Wilfrid Laurier University Research Ethics Board and written informed consent was obtained from each participant.

### Participants

Seventy students (graduate and undergraduate) participated in a screening session in a lab setting. Inclusion factors consisted of right-handedness, physical fitness and normal or corrected-to-normal vision. Exclusion factors included claustrophobia, pregnancy, regular smoking, extensive damage to the right hand, and medical conditions that could cause a health risk in the MRI environment (cardiovascular problems, electronic implants, back problems, or injury by a metallic object that was not removed). Of the original group of 70 subjects, 19 were chosen to participate in an fMRI scanning session based on their ability to perceive the illusion (4 males and 15 females). One male and 3 female subjects were excluded from this analysis due to a failure to experience the illusion sufficiently during the scanning session. Data was therefore analyzed for a total of 15 subjects: 3 males and 12 females with a mean age of 21.6 years.

### Apparatus

**Figure 1:**
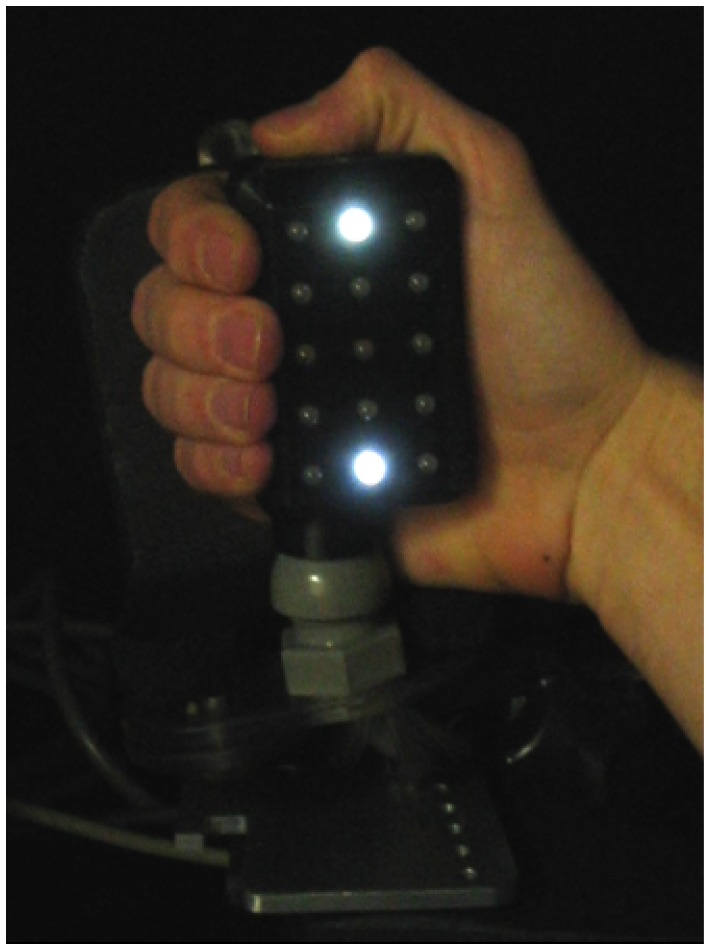
Photograph of the visuotactile stimulation apparatus.

We measured the BOLD (blood-oxygenation level dependent) response using fMRI while subjects viewed either one or two flashes of a light paired simultaneously with two pulses of air on their right hand using a custom-built apparatus that contained a 3×5 matrix of LEDs and a 3×5 matrix of pneumatic jets on opposing surfaces (see Figure 1). This device was attached to a fiberglass cradle in which subjects placed their right hand. The middle LED in the top row of the matrix served as a fixation point and the middle LED in the bottom row served as a target light. These two lights were 5.5 cm apart, and a chin rest was used to keep the viewing distance constant at 45 cm during the screening session to maintain a visual angle of 7.0° between the lights. The pneumatic jet directly opposite the target light delivered task-irrelevant pulses of air to the palmar surface of the right hand. Subjects responded using their left hand which controlled two plunger-style buttons (corresponding to one or two perceived flashes). The labeling of the two buttons (as 1 or 2) was counterbalanced across subjects. The stimulus apparatus and the buttons were connected to an IBM ThinkPad computer via a USB port. Software written in Matlab (Version 7.1; *MathWorks*, Natick, MA) coordinated stimulus presentation and recorded behavioural data. In the screening session, earplugs and headphones playing white noise were used to muffle the sound of the pneumatic jets.

During the scanning session, the stimulus-presenting device was firmly fixed on to the base that slides inside the MRI machine. Subjects lay in a supine position with their right arm in the fiberglass cradle, situating the stimulus-presenting device above the subject’s torso. A double mirror was placed above the subject’s head allowing a view of the LEDs on the device. The viewing distance was approximately 45 cm and the response buttons were located in the subject’s left hand.

### Stimuli

The upper fixation light remained on for the entire duration of each trial (1200 ms). The target visual stimuli either consisted of one 402 ms flash of the target light or two 201 ms flashes of the target light 9 ms apart. The visual stimuli were made to be less reliable than the tactile stimuli by using very short inter-stimulus intervals; the tactile stimuli consisted of two 70 ms air pulses with 140 ms inter-stimulus intervals (air pressure set to 30 psi). When one flash was paired with two pulses of air, the midpoint of the flash coincided with the midpoint of the tactile inter-stimulus interval. When two flashes were paired with two pulses of air, the midpoints of the flashes were aligned with the midpoints of the air pulses. The middle of the stimuli duration was normally randomized to occur within 333 ms of the middle of the trial.

### Procedure

**Figure 2:**
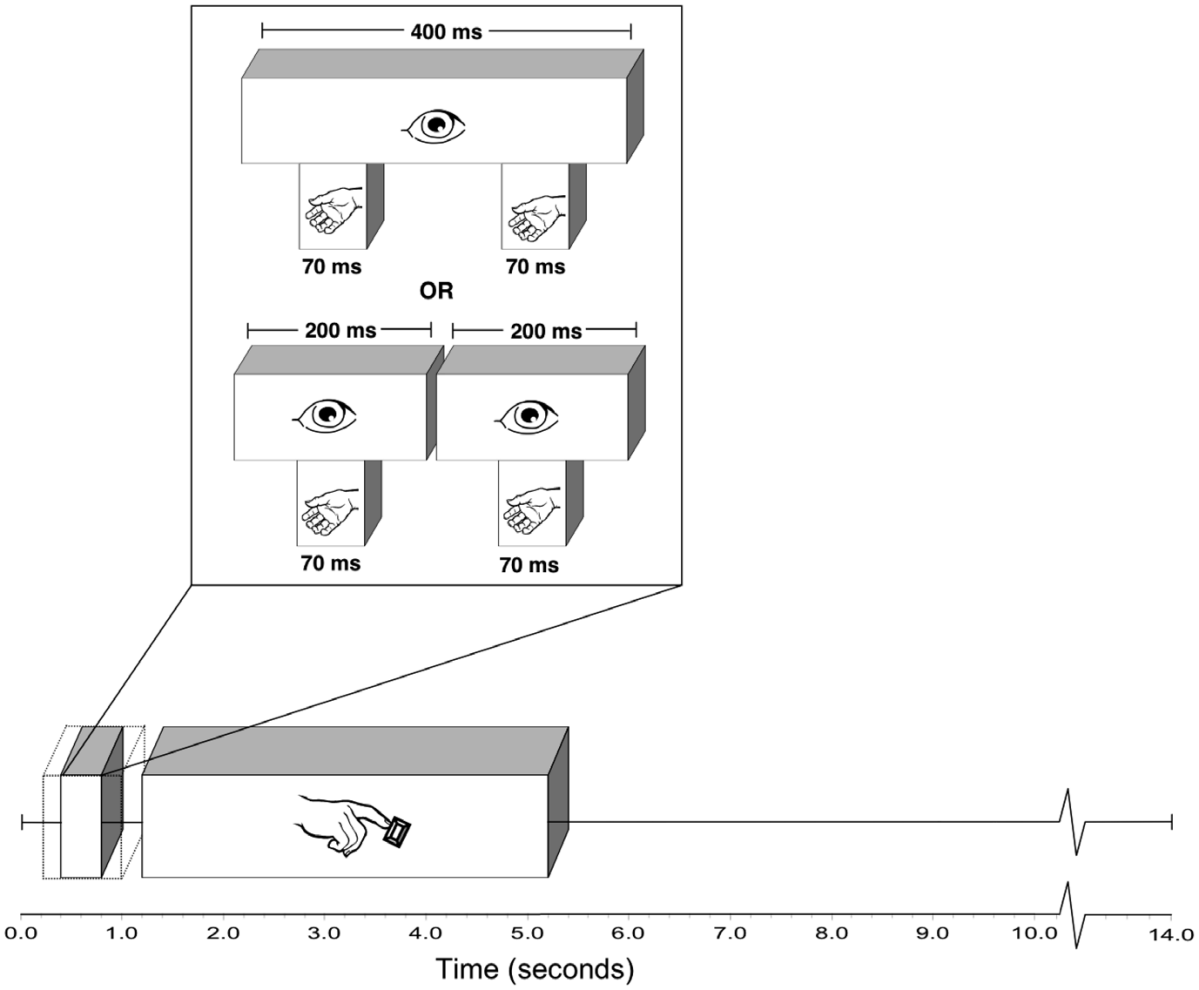
Overview of the event-related design used during scanning session.

Prior to imaging, a screening session was held at Wilfrid Laurier University to identify subjects who would be suitable for the fMRI session. Initially, subjects were evaluated for handedness using the Edinburgh Handedness Inventory [9] and eligibility criteria. In both the screening session and the scanning session, the trials had the same structure; subjects observed either one or two flashes of the target light presented concurrently with two pulses of air to their palm. Following each trial, subjects indicated whether they perceived one or two flashes via a button press during the inter-trial-interval. During the screening session, this inter-trial-interval had a variable duration and ended once the subject provided a response. The scanning session had an event-related design, with inter-trial-intervals of 12.8 seconds (see Figure 2). Each of the three blocks of trials contained 18 one-flash trials and 9 two-flash trials in random order, for a total of 54 one-flash trials and 27 two-flash trials. The screening session consisted of one block of practice trials as well as three blocks of experimental trials, with an opportunity to rest between blocks. This block of practice trials consisted of 12 one-flash trials and 6 two-flash trials. Subjects were selected for the scanning session based on their frequency of responses in specific conditions (at least 14 responses in each of the following three categories: one flash reported as two, two flashes reported as two, and one flash reported as one). This standard was used because the maximum number of useable trials for each condition is 27 (if a subject was to report experiencing the illusion on exactly 50% of one-flash trials), and the cutoff criterion represents one half of the maximum number of trials.

**Figure 3:**
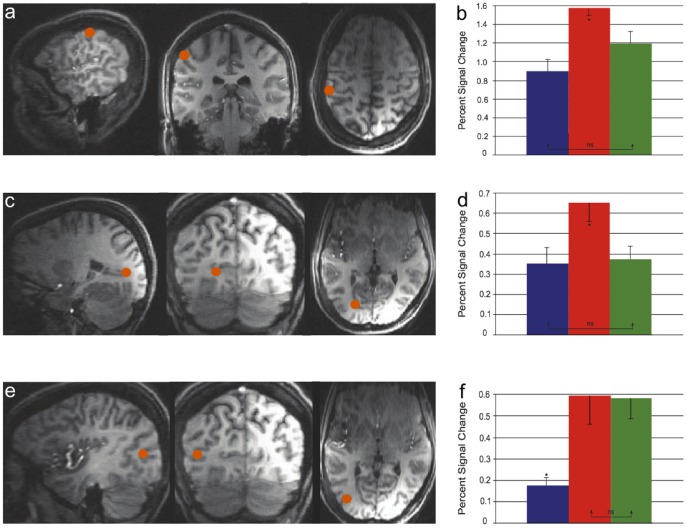
Centroids of regions that modulated their activity in response to the touch induced visual illusion within (A) the left IPS, (C) the left lingual gyrus, and (E) the left middle occipital gyrus. All three panels from left to right display sagittal, coronal, and axial views. Mean percent signal change plots for these regions are shown in (**B**), (**D**), and (**F**). Blue bars: no illusion trials (one flash reported as one flash); Red bars: illusion trials (one flash reported as two flashes); and Green bars: no illusion trials (two flashes reported as two flashes).

The scanning session was held at Robarts Research Institute (London, ON). The design during this session was identical to that of the screening session, however there were only six practice trials and there was a minimum of three blocks of experimental trials, with a maximum of five blocks. Three of these six practice trials ended once the subject provided a response, and the remaining three had inter-trial-intervals of 12.8 seconds, to familiarize subjects with the timing between trials in the scanning session. During the experimental trials, the number of responses in each of the three categories of interest were monitored following each block of trials to determine how many blocks were required; additional blocks were run until at least 14 responses were made in each of the three following categories: one flash reported as two, two flashes reported as two, and one flash reported as one. Four of the nineteen subjects failed to achieve 14 responses in at least one of the three categories and were not included in the subsequent fMRI analyses. The remaining 15 subjects, on average, required 4.2 blocks of experimental trials to achieve criteria. During scanning, subjects wore earplugs and were asked to push the buttons only with their left index finger. Response time was collected during scanning runs.

On about 30% of the one-flash trials subjects reported two flashes. In behavioural pre-testing and in a debriefing session after the fMRI session, subjects’ reported phenomenology was such that they were unable to distinguish between illusion trials (i.e., trials in which only one visual flash occurred) and trials in which two visual flashes were presented.

### Image Acquisition

Images were acquired using a 4.0 Tesla Siemens/Varian whole body imager with a custom-built ‘clamshell’ transceive surface coil [10] for enhanced signal in temporal, parietal, and occipital cortex. During the experimental trials, T2*-weighted segmented-echo-planar images were obtained (19 coronal slices (spanning occipital pole to precentral gyrus), 4.0×3.0×3.0 mm, FOV: 192 mm), with a matrix size of 64×64 and total volume acquisition time of 2 s. Following the functional scans, a ten-minute T1-weighted scan collected anatomical images (96 2.0 mm coronal slices), with the same field of view and a matrix size of 256×256.

### Analysis

FMRI data were analyzed for each of the following behavioural categories: one flash reported as two (illusion trials), one flash reported as one (one-flash non-illusion trials), and two flashes reported as two (two-flash non-illusion trials).

Across runs, a total of fourteen trials for each behavioural category were chosen per subject. The first step in the trial selection process involved discarding all trials with response times greater than 4 seconds. For a given subject, trials were first chosen for the behavioural category with the lowest frequency of responses, giving preference to trials that occurred closer to the beginning of a run (to minimize effects of linear drift and motion artifacts). Trials were then chosen for the remaining two conditions, with care to closely match the temporal distribution of trial types over the duration of the experiment. For each subject, a one-way Analysis of Variance (ANOVA) was conducted on the dependent variable response time. If response time was found to be significantly different across the three behavioural conditions, the trial selection was revised until there was no significant difference across conditions.

Multiple time courses were created for each subject by selecting the trials of interest from each run. For each run containing trials selected for analysis, a separate time course was created for each of the three following conditions by extracting the relevant functional data: illusion trials (one flash seen as two), two-flash non-illusion trials, and one-flash non-illusion trials.

Using BrainVoyager QX software (Brain Innovation), 3D motion correction and linear trend removal were performed on the functional data. These data was then co-registered with each subject’s corresponding anatomical images, which were isovoxelled and transformed into Talairach space [11]. Functional data for each condition was then correlated with the reference time course which was shifted according to the hemodynamic response function (HRF). For each condition, activated areas were defined by running a random effects general linear model (RFX GLM) analysis, and by overlaying each of the three different contrasts. For each contrast, a threshold of *p*<0.01 was used for two or more contiguous voxels. Cluster size was noted and percent signal changes for each condition were recorded.

## Results

Three cortical regions modulated their activity (p<0.05, corrected) in response to the illusion: left intraparietal sulcus (IPS), left lingual gyrus, and left middle occipital gyrus with Talairach co-ordinates [11] of respectively −56, −29, 47; −23, −73, 2; −41, −73, 4 (see Figure 3). The regions within the IPS and the lingual gyrus did not differentiate between one-flash non-illusion trials and two-flash non-illusion trials (both comparisons p>0.5). In contrast, the region within the middle occipital gyrus differentially modulated its activity during the one-flash non-illusion trials as compared to the one-flash illusion trials and the two-flash non-illusion trials.

## Discussion

Regions within the IPS and the lingual gyrus increased their neural activity on trials in which subjects experienced the illusion relative to when they did not (presented with one flash and reported one). Because this visual illusion is driven by tactile information this suggests that these areas receive information from the somatosensory system. That the IPS receives tactile information is not surprising given a large body of work implicating the IPS in multisensory processing including signals from the visual and somatosensory systems [12]. Indeed, neuroimaging studies of visuo-tactile illusions such as the rubber hand illusion consistently show that the IPS plays a role in somatosensory-visual binding [2], [13]. The visual functions of the lingual gyrus are also well known [14] and there is some neuroimaging evidence that it may also receive somatosensory inputs [15]. The enhanced activation in the IPS and lingual gyrus during the illusion trials relative to non-illusion trials (both one-flash and two-flash trials) is consistent with the idea that binding of the visual and somatosensory streams occurred – such signal enhancements are known to occur in multisensory integration tasks [16]. Interestingly, activity in these two regions was enhanced during the illusion trials even when compared to the veridical two-flash trials. One might speculate that these brain regions are working harder to make sense of two streams of discrepant sensory inputs arriving at the same time and somehow resolve this by ‘fabricating’ a second visual event to be consistent with the second tactile event. One could further speculate that such an effect might be driven by top-down influences via recurrent projections as has been described for the visual system [17], [18].

In contrast to the IPS and lingual gyrus, the region within the left middle occipital gyrus was more active when subjects responded ‘two’ (for either illusory or non-illusory perceptions) compared to when they responded ‘one’ (non-illusory perceptions only). In other words, the middle occipital gyrus does not differentiate between one-flash illusion trials and two-flash trials. They are both treated as a ‘two’ response. What this region seems to code for then is the decision to respond ‘one’ or ‘two’. Intriguingly, some work suggests that the middle occipital gyrus might be involved in subitizing and counting [19].

Modelling perceptual decisions in non-human primates has been an extremely fruitful enterprise. The key features of such models is that they posit a cortical region that contains sensory evidence and a separate cortical region that contains the decision variable [8], [20]. Two sensory paradigms in particular have provided extensive evidence for this idea: direction of visual motion discrimination [6] and tactile frequency discrimination [7].

In the direction of motion visual discrimination task cells within area MT provide the sensory evidence and cells further downstream such as those within area LIP form a decision by computing the difference between the activities of populations of neurons in area MT that code for opposite directions of motion [6]. Likewise in the tactile frequency discrimination task the sensory evidence is provided by area S1 and the decision variable is computed by cortical regions further downstream such as area S2 [7].

The present study provides evidence for the first time that such parcellation of function exists in the human brain as well with the regions within the IPS and lingual gyrus representing sensory evidence and the region within the middle occipital gyrus accumulating sensory evidence to compute a decision variable. Why we observe two regions that appear to represent the sensory evidence is unknown although, certainly in the visual motion and tactile frequency paradigms previously described in the monkey, there is more than one cortical region that represents the sensory evidence and decision variables [20].

What factors determine whether or not a subject succumbs to the illusion in our study is still an open question. Weighting rules related to how the two sensory streams are combined [21], attention [22], and neural noise [23] may all play a role.
